# Auditory Mismatch Negativity Under Predictive Coding Framework and Its Role in Psychotic Disorders

**DOI:** 10.3389/fpsyt.2020.557932

**Published:** 2020-09-10

**Authors:** Chun Yuen Fong, Wai Him Crystal Law, Takanori Uka, Shinsuke Koike

**Affiliations:** ^1^ Center for Evolutionary Cognitive Sciences, Graduate School of Art and Sciences, The University of Tokyo, Meguro-ku, Japan; ^2^ Department of Integrative Physiology, Graduate School of Medicine, University of Yamanashi, Chuo, Yamanashi, Japan; ^3^ University of Tokyo Institute for Diversity & Adaptation of Human Mind (UTIDAHM), Meguro-ku, Japan; ^4^ University of Tokyo Center for Integrative Science of Human Behavior (CiSHuB), 3-8-1 Komaba, Meguro-ku, Japan; ^5^ The International Research Center for Neurointelligence (WPI-IRCN), Institutes for Advanced Study (UTIAS), University of Tokyo, Bunkyo-ku, Japan

**Keywords:** prediction, MMN, mismatch negativity, schizophrenia, predictive coding

## Abstract

Traditional neuroscience sees sensory perception as a simple feedforward process. This view is challenged by the predictive coding model in recent years due to the robust evidence researchers had found on how our prediction could influence perception. In the first half of this article, we reviewed the concept of predictive brain and some empirical evidence of sensory prediction in visual and auditory processing. The predictive function along the auditory pathway was mainly studied by mismatch negativity (MMN)—a brain response to an unexpected disruption of regularity. We summarized a range of MMN paradigms and discussed how they could contribute to the theoretical development of the predictive coding neural network by the mechanism of adaptation and deviance detection. Such methodological and conceptual evolution sharpen MMN as a tool to better understand the structural and functional brain abnormality for neuropsychiatric disorder such as schizophrenia.

## Introduction

From an evolutionary perspective, the nervous system of animals needs to be highly adaptive to the constant changes of the physical world to increase their survivability. To achieve this, an accurately perceived sensory input and the respective cognitive processing, as well as an effective interaction between them are needed. The animal brain, therefore, forms a dynamic network linking its own body and the external environment (1). This perspective becomes fundamental to many contemporary brain-behavioral models. Perceptual learning, for instance, highlights how our sensory system is shaped and tuned by past experiences. Such exceptional ability allows animals to filter off unwanted sensation (noise) and concentrate on the unexpected information, based on their prior knowledge. This function is termed as prediction in predictive coding theory of the brain, serving as one of its foundational concepts (2–4).

**Figure 1:**
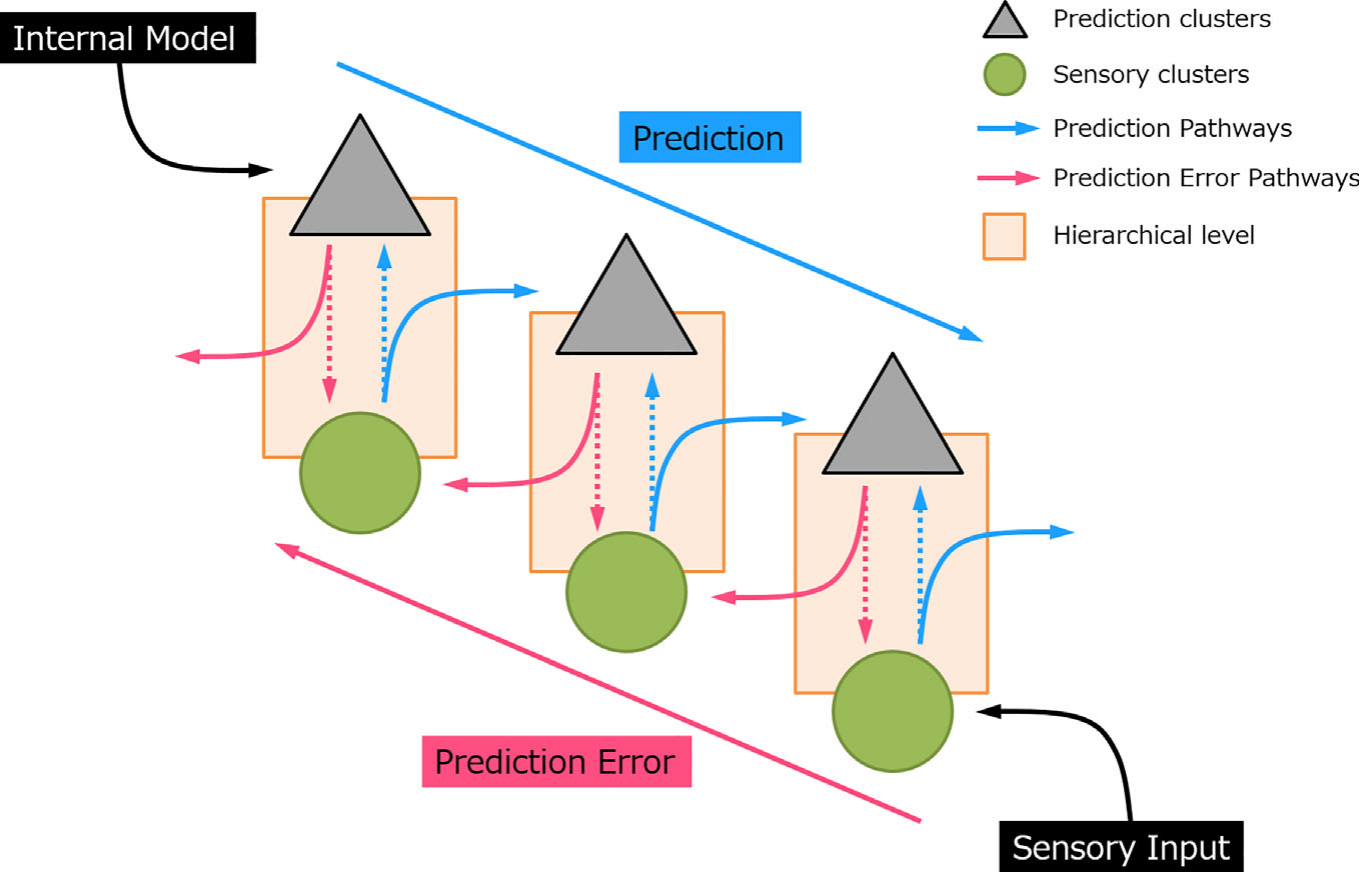
A simplified sensory prediction model. The blue arrow indicates how prediction is updated on the prediction neurons and pass to a lower hierarchical level. The red arrow indicates how prediction error is generated and feedforward to a higher hierarchical level. Each hierarchical level could represent a processing layer, for example, in auditory processing, i.e., auditory cortex, thalamus (medial geniculate body), and midbrain (inferior colliculus), respectively (from top to bottom).

Predictive coding has gained increasing interest in recent decades and appears as a robust underlying mechanism of perceptual learning (3, 5). The notion of predictive coding suggested that the brain continuously generates and updates its predictions to the physical world by integrating the sensory information. In this manner, perception is not a uni-directional projection, but a bi-directional and hierarchical process (see **Figure 1**). In sensory processing, sensory “prediction” is actively generated by an internal model at the higher-order area which incorporates information from the past (memory), produces and sends information regarding the future to the lower hierarchical cascade. This inferential process naturally allows the implementation of the Bayesian theories, where prior sensory experiences and current sensory input were used to compute the posterior perceptual estimation, that is, the prediction (6–8). At each processing layer, neurons subtract this prediction from the sensory data and register the residual by a “prediction error” signal. In this sense, it is not necessary for an expected event to transmit to the top of the cortical hierarchy (9). Instead, only the information that deviates from predictions is further processed and passed upward to the higher-order cortical area in the form of “error”. The greater the prediction error, the larger the neural response evoked. This prediction error signal propagates through an ascending pathway and updates the subsequent prediction. When the prediction eventually matched with the sensory input, the neural activity induced by prediction error would be suppressed.

While it may seem easy to assume that one is consciously aware of the role of prediction in everyday decision making (10), it is neither fair nor true. There are many “less obvious” predictions hidden along the sensory system in one’s daily life that are likely to go unnoticed. For example, a goal-directed behavior as simple as grabbing an object first requires the integration of predictions of the visual and interoceptive inputs to ensure the precision of the sensory information, such as the locations of the object and our hand [see (11)]. With these accurate sensory inputs, the brain can produce the corresponding muscle movements *via* the corticomuscular channel as an end-result. Some literature showed that this succession of neuronal activities can be modulated by the dopaminergic reward system (12, 13).

With such a fascinating theory on sensory perception, scientists put effort into “visualizing” or quantifying prediction and prediction error in order to justify their proposed neural mechanism. This is later achieved by experiments that initiate prediction by the repeated presentation of certain stimuli at a given rhythmic time interval and subsequently stop delivering the predicted stimulus. This paradigm generates a transient neural response in the absence of sensory input, which becomes strong evidence of sensory prediction. For example, by repetitively presenting auditory or visual cues followed by a button press (motor response), the enhanced event-related potential (ERP) could be evoked by the button press even without the actual feedback stimuli (14–16). Using a similar technique of attributing sensory feedback with another sensory cue, pre-activation of the sensory cortex was also observed *via* functional imaging which is in support of the subjective prediction in sensory input (17).

The notion of predictive coding has been adopted and confirmed by a wide range of neurocognitive computational models (2, 7, 18–21). Its extensive application also provides valuable insights into the investigation of many psychiatric and developmental disorders such as schizophrenia and autism (22–24). For instance, abnormalities (hyper/hypo-responsivity) in sensory processing were hypothesized to be responsible for several symptoms of psychiatric disorders (25). Disruption of the exteroceptive prediction may lead to visual and auditory hallucinations while the disturbances of interoception might also give rise to emotional disorders (20, 26, 27). In this paper, we seek to review the neural basis and empirical findings of sensory prediction under the predictive coding framework. The majority of experimental and clinical evidence for sensory prediction was provided by the mismatch negativity (MMN) paradigm in which the prediction error fluctuation underlies the breaking of a stream of acoustic sounds. Therefore, we seek to give an extensive review to such task and discuss its application in neuropsychiatric disorders using the example of schizophrenia.

## Sensory Prediction in the Visual System

If we look around the living room where we are staying in, we would not be surprised to see objects such as chairs, desks, lamps, or windows. However, the presence of a wild animal or a stranger in the room would undoubtedly shock and alert us. Predictive coding theory suggested that the higher cortical area would constantly produce internal models of the surroundings and make expectations on what objects we would see. This information would be passed to the lower order neural structure, forming predictions on visual features as simple as colors and shapes (28). In this example, assuming that we live alone, the prediction error is generated at the hierarchical level where we identify the objects we see as human or animal (which are objects that we do not expect to see in our living room). On the other hand, if we live with our family and pets rather than alone, the upward propagated prediction error might appear later at a higher level when our brain successfully identifies those objects as “intruders”. This suggested visual perception is not only driven by the bottom-up sensory input but also influenced by the top-down prediction model (29).

The predictive ability in the visual system can be observed as early as in the retina. Previous studies showed that retinal ganglion cell had enhanced sensitivity to unpredicted changes and suppression for predicted stimulations in various domains of visual stimuli including orientation, spatial frequency, and temporal structure (30). This property of receptive fields is consistent with the notion of predictive coding. At the lower hierarchical level, prediction of the visual system can also be seen from salience detection, which is guided by a “centred-surround prediction” mechanism (3, 31). In a simple visual search task, a salient visual feature (e.g., color, motion, and orientation) can be spotted irrespective to the number of surrounded visual distractors (32). Since a pixel in the natural scene tends to correlate with the neighboring pixels, the brain is able to form a spatial prediction on the centred pixel by the weighted linear sum of the surroundings (3). A salient stimulus is inconsistent with the internal computational model, which produces an error detection signal that increases along the visual hierarchy *via* retina (33, 34), lateral geniculate nucleus [LGN; (35)], striate and extrastriate cortex (3), and lateral intra-parietal cortex (36). This signal passes along the visual hierarchy, reaches the oculomotor system, and re-orients the eye-gaze to the location with high salience (37). Such hierarchical communications are well-supported by the feedforward and feedback connections in the cortico-cortical and subcortical-cortical network which have been described in detail in previous literatures (38–40).

The predictive power of the visual system also compensates the limited capabilities per se. For example, human retina can only encode two-dimensional information. In addition to the retinal disparity produced by our two visual fields, visual experiences such as an object’s relative size and aerial perspective also contribute to our 3-D vision by providing the perception of depth. Besides, the number of neurons and energy required for metabolic activities restrict the computational power (e.g., sampling rate and speed of signal transmission) of the visual system. Visual prediction, therefore, becomes crucial in motion perception. By sampling an object’s movement within hundred milliseconds, the visual system is capable to predict the motion pathway of the object which provides the visual ability to pursue an object moving in constant velocity (41–43).

A more active form of visual prediction happens when we voluntarily focus on a location in space and enhance our visual sensitivities on that specific location. This ability to guide our spatial attention is based on one’s prediction on where a task-relevant cue might be presented. Eye movement signals in frontal eye field (FEF) can also enhance the sensory gain in the visual cortex [for example, area V4; (44, 45)]. Previous studies have shown that neuronal activity can be modulated by the prediction of stimulus onset (46, 47). By conditioning a visual target on a certain location with a sensory cue after a period of time, a monkey could learn the conditioned patterns of the visual events by making eye movements to the target with a precise temporal pattern (47). An increased neuronal response could be observed from their parietal cortex when the visual target was absent from the conditioned location unexpectedly. This paradigm is similar to the terminology of the oddball experiment where a “deviant” event elicits an elevated neural response, which is also known as MMN, compared to the “standard” (48). This visual MMN signal can be suppressed by silencing the somatostatin-containing GABAergic interneurons (SOMs) pharmacogenetically (49).

Overall, the empirical evidence of the visual system appears to support the notion of predictive coding. In the following sections, the sensory prediction in the auditory system would be described in more detail.

## Sensory Prediction in the Auditory System and Mismatch Negativity

Similar to the visual counterpart, the auditory prediction is vital to cognitive functions, including language, and speech processing (50). In spoken word recognition, it has been demonstrated that there are top-down predictions in the lexical-semantic system. For example, studies showed that replacing the last syllable of a word such as “formula” to “formubo” can facilitate an enhanced evoked response at superior temporal gyrus (STG) on the listeners (51). Another experiment showed that a non-Finnish language vowel presented in a train of Finnish vowels could elicit a pre-attentive neural response at the left auditory cortex (AC) (52). Predictive coding hypothesized that these neural responses originated from the prediction error signal when the expected vowels unmatched with the actual acoustic signal somewhere in a higher cortical area of the linguistic hierarchy. Music perception was also hypothesized to be inferred by our experience and knowledge on music (e.g., melodic, temporal, and harmonic structure) while we are listening to a stream of musical tones (53).

The neural basis of prediction and prediction error, as well as the evidence of the predictive coding hierarchy in the auditory domain, was explored most extensively by the studies of auditory MMN. The MMN is a negative deflection of the ERP elicited by an abrupt change of auditory stimulus after the repetition of the same sound, indexing the pre-attentive sensory processing (54). MMN is not a manifestation unique to the auditory modality. It can also be elicited by visual stimuli using a similar experimental setup [for more detail, please see the reviews of vMMN from (55) and (56)]. MMN reduction is one of the most robust biomarkers in patients with schizophrenia (57). Therefore, the neural mechanism underlying MMN has raised significant interest over the years.

The MMN in a classical oddball paradigm was measured by electroencephalogram (EEG) when an infrequent deviant (d) sound was interspersed among a train of repetitive standard (s) sounds (e.g., s s s s s s d s s s s s d s s s …) (58). The deviant tone can be different from the standard in terms of frequency, duration, or intensity. Traditionally, the MMN is obtained by subtracting the event-related response evoked by the standard tone from the response of deviant tone. MMN experiment can be carried out in the absence of attention, though some studies showed that the MMN response is attenuated without the involvement of attention (59).

## Neural Basis of Mismatch Negativity Under the Predictive Coding Framework

The auditory MMN often peaked at the frontocentral scalp that inverts polarity at the posterior-temporal regions (60). Dipole analyses on EEG and magnetoencephalogram patterns revealed that the neural generator of this response was located at the AC which lies along the STG in each hemisphere (61, 62). Whether the deviant was presented as a mismatch in frequency, duration or intensity shifted the dipole source over the supratemporal cortex (63–66). Other regions have also been reported to contribute to MMN in an auditory oddball paradigm, for example, frontal cortex [FC; (67–71)], subcortical structures such as thalamus (21, 72, 73) and hippocampus (74, 75). Despite decades of clinical and experimental research, the role of different neural structures and the actual neural mechanism underlying MMN remained controversial. Two main hypotheses have been most extensively studied, namely, “adaptation” and “sensory memory/predictive coding” (76).

**Figure 2:**
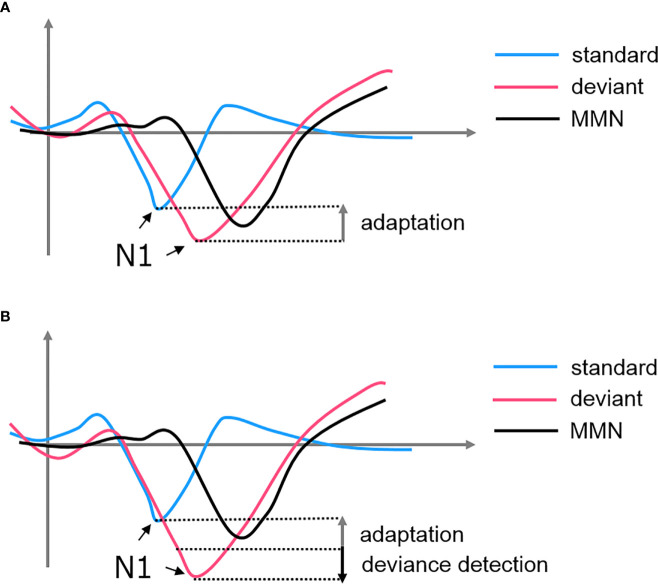
The formation of MMN from the subtraction of standard-evoked potential from deviant-evoked potential. **(A)** SSA hypothesized that the MMN is simply a derivative of the attenuated N1 of the standard. **(B)** Predictive coding hypothesized that both adaptation of the standard and deviance detection of the deviant contributes to the MMN.

Adaptation, also known as stimulus-specific adaptation (SSA), was interpreted as a lower-level inhibitory neural mechanism in response to repetitive stimulations (77). This property was observed at cortical and subcortical structures such as the AC, thalamus (medial geniculate body, MGB), and midbrain (inferior colliculus, IC) [see (72, 76–78)]. Some researchers argued that there is a bottom-up thalamocortical network where the AC is modulated by a synaptic depressive input projected from the thalamus (77, 79). In this case, MMN is a derivative of the N1 suppression when we subtracted the standard-evoked potential form the deviant-evoked potential. The subsequent negative deflection of the deviant signal simply represented a non-adapted evoked response, highlighting a “release” from the suppression (see **Figure 2**). However, this hypothesis failed to explain the MMN findings in some oddball experiments. For example, if the deviant in an oddball paradigm is replaced by an omission stimulus, the absence of the expected tone is still able to induce an MMN when no adaptation could have occurred (80, 81). Additionally, using a local/global paradigm, studies showed that the violation of local and global rules would elicit two different MMN signals at the AC and FC, respectively (82). These findings suggested that there must be a higher-order process encoding the patterns of sequence and producing an enhanced response when the encoding rule is violated (83).

**Figure 3:**
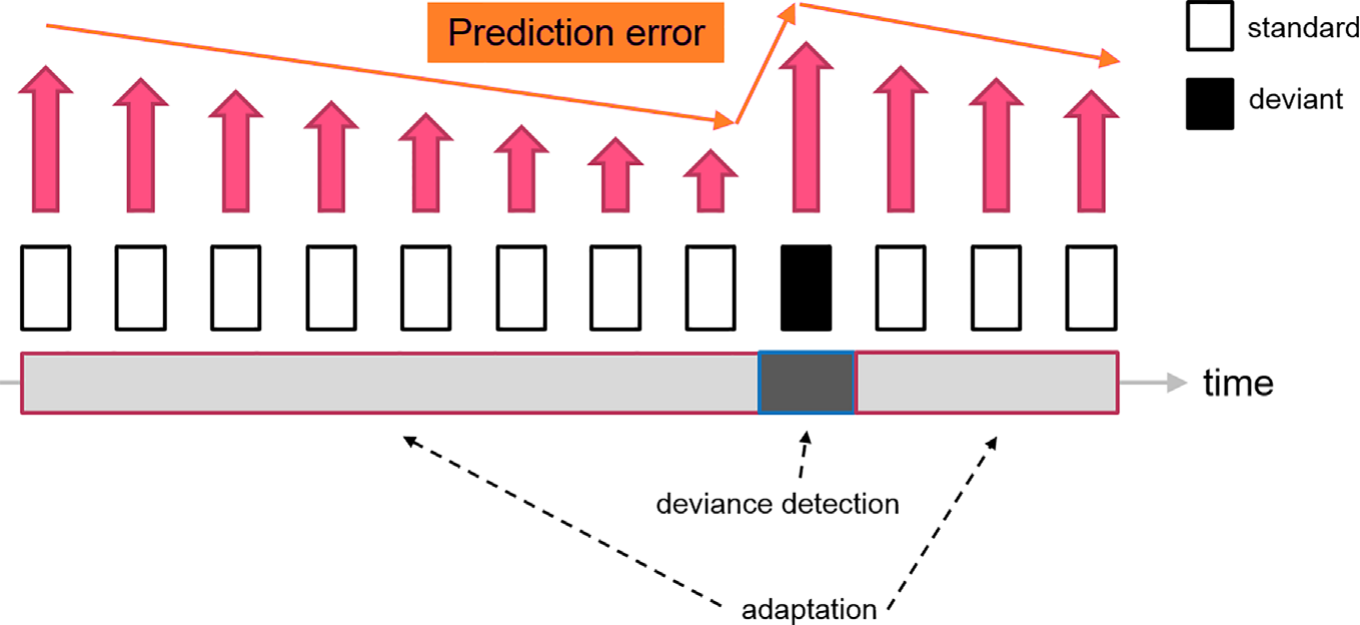
A diagram indicating the change of prediction error signal during a classical oddball experiment. The repetitive presentations of the standard stimuli reduce the prediction error signal (decrease neuron firing) feeding to the higher-order area, which leads to the “adaptation” effect. The subsequent deviant tone elevates the prediction error, which generates the deviance detection input (increase neuron firing). Therefore, the resultant MMN (deviant - standard) consisted of two neural mechanisms driven by the fluctuation of prediction error signals.

In contrast, predictive coding suggested that neurons in the thalamocortical network have a more sophisticated cognitive processing to gather sensory information. The standard stimulus, which is a series of repetitive sounds/tones, would be encoded as a sequence of patterns by the brain, forming a “prediction” for the upcoming stimuli. When the sensory data is matched with the prediction, the prediction error is minimized, leading to the neural adaptation. Since the prediction has been shaped by the standard stimuli, the deviant as a distinctive sensory input would produce a considerable amount of prediction error. The MMN is, therefore, an electrophysiological marker reflecting the level of prediction error when an unexpected stimulus (deviant) is presented (see **Figure 3**). As a limitation, this simplified model did not address the firing patterns of the prediction neurons. Therefore, the “prediction error signal” might indeed represent the ratio between prediction error and prediction signal or the net electrophysiological activities of these two clusters of neurons.

The prediction signal might only be visible as N1 during the adaptation period when the prediction error is theoretically minimized. With an early latency (peaked around 100 ms), adaptation is expected to be localized at the lower level of the prediction hierarchy formed by the subcortical-cortical network, involving mainly the belt area of MGB and IC with both the belt and core areas of the primary AC (A1) (84, 85). Previous studies revealed that the AC can have both excitatory and inhibitory effects on the MGB (86) and IC (87). Thus, the AC can suppress the predicted information from approaching the cerebral cortex by inhibiting the activities of the thalamus and midbrain when prediction error input is reduced (84, 88).

In addition, neurons at the IC with strong SSA properties appeared to receive extensive inputs from the AC while those with no/less SSA properties mainly receive input from the brainstem nuclei (89). Studies using a cooling technique to deactivate the cortical activity on animals also discovered that the SSA properties of certain neurons at the IC and MGB would be reduced while certain proportions of neurons still retained their SSA sensitivity (72, 84). Collectively, adaptation as a key component of predictive coding and MMN, appeared to be modulated by a lower order AC-MGB-IC network which continuously interacts with each other, maintaining the efficiency of sensory processing.

Deviant detection (DD) is another core component of the MMN signal. Using single-neuron recording, Parras, and colleagues demonstrated (90) on anaesthetized rats that the AC-MGB-IC network contributes to both the adaptation and deviance detection of the MMN signal. The prediction error activities elevated from IC to MGB to AC and from lemniscal to non-lemniscal regions. Other subcortical regions such as the cerebellum (91) and basal ganglia (92) were also known to be associated with the sensory prediction processing. However, DD has mainly been linked with the higher-order processing in predictive coding based on the temporal profile of the MMN, where the lower order response activates earlier (~100 ms) at the AC while the higher-order DD signal becomes visible in a later time window at the frontal area [100–200 ms after the stimulus onset; (93–95)]. The P3a followed by the frontal MMN also suggested that there could be a switch of attention toward the deviance (96).

Additional studies revealed that deviance detection was associated with the higher cortical area. An electrocorticographic (ECoG) study showed that deviance detection signals in the cerebral cortex are localized to the lateral STG and FC in humans (97). Other studies argued that the PFC (e.g., ventral prefrontal regions) contributed to DD when the sensory input projected from the AC (anterior belt) does not match the existing memory (98). The role of the frontal region in this sort of feedback mechanism is well-supported by the studies of non-conscious patients. For instance, patients in the vegetative state showed a reduction in feedback modulation with the sensory cortex while their feedforward functioning was still maintained (99, 100).

To sum up, predictive coding provided a comprehensive explanation to the temporal and spatial profile of MMN by the fluctuation of the prediction error signal, which was modulated by the cortical and subcortical network in a hierarchical manner. Associating adaptation and DD with lower-order regions and higher-order regions respectively were tentative, with no strict boundary between each neural hierarchy. Intuitively speaking, it seems logical to think that adaptation serves the function of gatekeeping at the early sensory pathway to prevent information overloading. Without excessive data input, the higher-order sensory cortex should perform the DD more efficiently. Since predictive coding assumes the same communication mechanism, formed by prediction and prediction error, for each neural structure, the functional specification of adaptation and DD might be achieved by the differences in neural properties and possibly the proportion of each type of the neurons along the neural hierarchy.

## Various Forms of Oddball Paradigm

**Figure 4:**
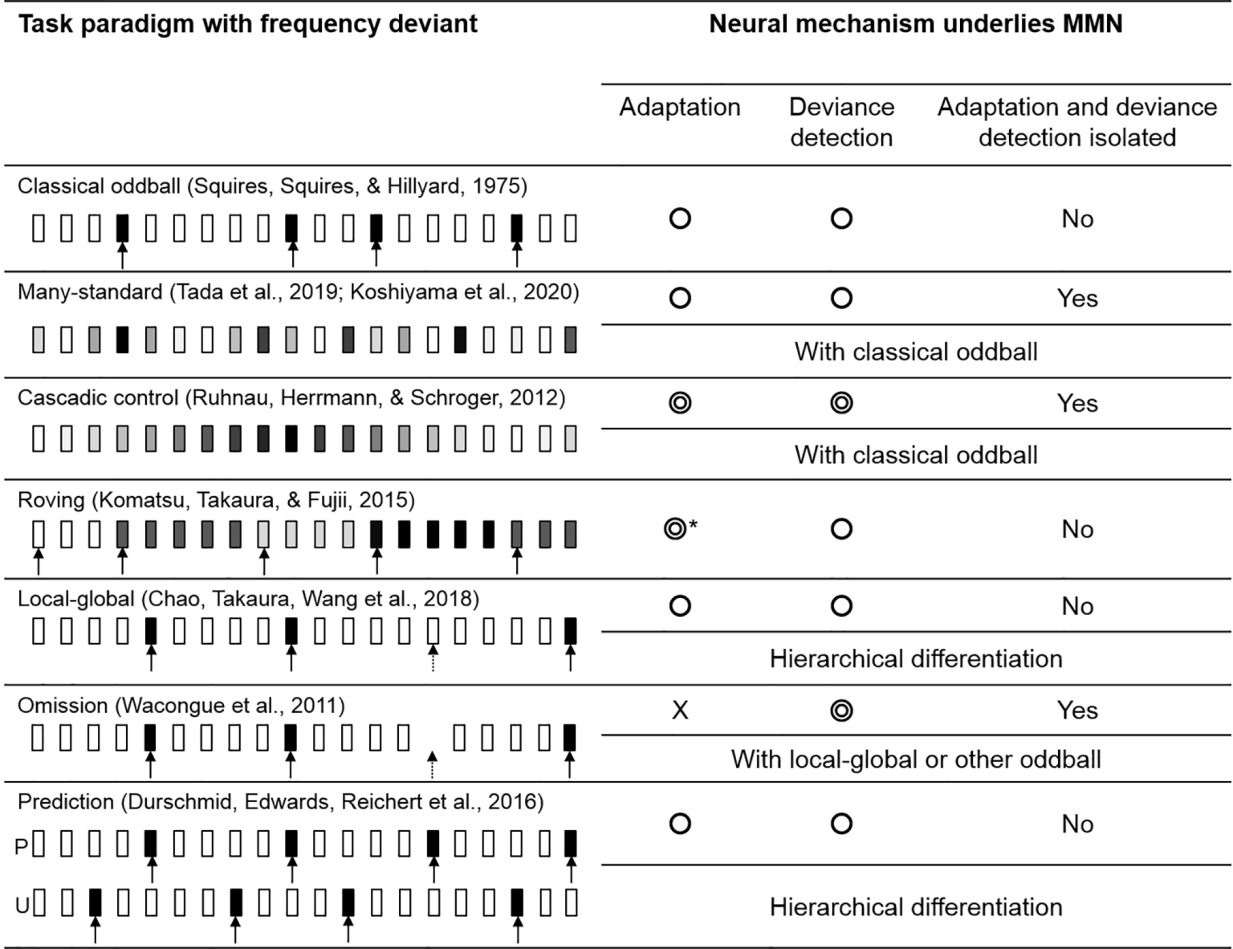
The variations of oddball paradigm. The circle symbols indicated whether the MMN would be influenced by adaptation or deviance detection. The double circle indicated that the MMN would give a cleaner representation of adaptation/deviance detection. Classical oddball coupling with many-standard and cascadic-control allows the isolation of deviance detection from adaptation. Without an actual auditory input, an omission deviant would elicit a signal that “free” from adaptation, while MMN in other paradigms would be influenced by both mechanisms. *Roving paradigm allows the comparison of MMNs evoked by different length of standards in which adaptation effect could be investigated. The greyscale indexed the pitch of the stimulus; the arrow indicated the deviant tone (the dashed arrow indicated the global deviant).

The deviant evoked response in a classical oddball paradigm is known to be influenced by both adaptation and deviance detection. By introducing various forms of oddball paradigm and control task, literatures found that the evoked response, more precisely speaking, could reflect the cognitive process of adaptation and deviance detection separately. In the following section, we briefly describe 7 major MMN paradigms and compare their effectiveness in identifying the underlying mechanism (see **Figure 4**).

### Classical Oddball

Traditionally, the MMN in the classical oddball paradigm is obtained by subtracting the standard-evoked potential from the deviant-evoked potential. However, the adaptation effect induced by the standard tone was known to cause a reduced auditory N1. By subtracting a standard evoked response with a reduced N1 (peaked at 100 ms) from the deviant evoked response (peaked at around 100–200 ms), the resultant MMN could be contaminated by the N1 and hence overestimated.

### Many-Standard Control

The many-standard paradigm was developed as a control paradigm to eliminate the adaptation effect (N1) from MMN. A simple “many-standard” control consisted of a sequence of tones [e.g., varied in pitch but fixed duration, loudness and onset-to-onset intervals (78, 101–104)]. Two of the tones were matched with the pitch of the standard and deviant tone using in a classical oddball respectively, while the rest of tones have a random pitch. A many-standard control presents the tones in pseudo-random order, creating a sequence with no known regularity which prevents the formation of adaptation among the participants.

Therefore, by comparing the event-related response evoked by the deviant tone in the classical oddball paradigm with the response elicited from the tone with the same pitch but presented in the many-standard sequence (without the reduced N1), researchers should be able to obtain the “adaptation-free” MMN that represents the deviance detection process. On the other hand, the comparison between the neural response evoked by the “standard” in classical oddball and the many-standard paradigm should identify neural signals that indicate the adaptation effect. This also allows the comparison on two identical tones, eliminating any confounding effect produced by the acoustic features (i.e., pitch for a frequency deviant) rather than the abstract rule of the sequence.

The disadvantage of the many-standard control is that the randomness of the sequence would generate a considerable amount of prediction error according to the predicting coding theory. Therefore, the comparison with the oddball sequence may give an underestimated deviance detection effect and an overestimated adaptation effect.

### Cascadic Control

With the limitation of a many-standard control, an additional sequence coupling with the classical oddball and many-standard paradigm, known as “cascadic-control” was introduced (105). The cascade sequence consisted of a regular train of tones in increasing or decreasing frequencies. The target control tone is hidden in a predictable sequence which suppresses the generation of prediction error from the control sequence. The descending or ascending cascade control corresponds to the oddball deviant that has a decreased or increased frequency respectively. The preceding tones of the control and oddball sequence are also identical, which makes the cascade a better control compared to many-standard to identify the deviance detection process.

### Roving

The roving paradigm contains multiple trains of tones in various frequencies (106–108). Each train consists of a random amount of identical tones separated by regular inter-stimulus-intervals. The repetition within each train of tones eventually leads to an adaptation effect; thus, the last tone of the block can be viewed as a “standard” as in the oddball paradigm. The first tone of a train, on the other hand, indicates a switch of frequency, making it a natural deviant tone with the same physical properties as the standard. Similar to standard oddball, the MMN is indicated by the comparison of standard and deviant evoked response. In addition, the roving paradigm can examine the build-up of the adaptation by comparing the MMN caused by the different numbers of repetitions between the deviant and standard tone (109).

### Local-Global

The local-global paradigm typically consists of two sets of auditory stimuli (82, 110). A set of tones, named as local standard, contains a series of identical tones (e.g., AAAAA). The repetition of tone A is the local rule of this sequence which makes the sequence AAAAA fitting the internal rule. Another set of tone, named as local deviant, also contains a train of identical tones but the last one is replaced by a different tone (e.g., AAAAB). Therefore, the internal rule of repeating tone A in this sequence is violated by the last tone. By presenting the local deviant repeatedly (e.g., AAAAB AAAAB AAAAB…), our brain can identify such sequence as a global-rule, which makes the local deviant a global-standard. If a rare number of local standard (AAAAA) is mixed randomly into the train of local deviant (e.g., AAAAB AAAAB AAAAB AAAAA AAAAB AAAAB AAAAB AAAAB AAAAB AAAAB AAAAB AAAAA AAAAB …), the local standard here then appears as violating the global rule, which turns the local-standard as a global-deviant. By interchanging the proportion (probability) of the local-deviant and local-standard, their roles in the global-rule can be swapped.

This paradigm introduced two types of temporal regularities, that is, the local rule (tone-to-tone transition) and the global rule (sequence-to-sequence transition). The detection of violating a local-rule was hypothesized as lower-order processing while the global-rule was hypothesized to be processed at the higher-order cortical area. This fits the paradigm into the predictive coding manner which proposes a hierarchical communication network. Chao and colleagues showed that the local prediction errors were generated in the AC while the global prediction errors were generated in the FC (82). Their ECoG connectivity analyses revealed that local prediction error signals were fed forward to the AC and that global prediction error signals were fed forward to the FC. A feedback component from FC to AC for global prediction errors was found as well.

### Omission

All oddball paradigms described above have both standard and deviant tones. An omission paradigm often couples with other oddball paradigms as an additional sequence where an omission deviant replaces the typical frequency deviant (110). In other words, the expected event is omitted (no tone) followed by a train of standard tones. Since no actual acoustic input is presented, the evoked response cannot be sensitive to the adaptation effect, which allows the isolation of deviance detection signal.

### Prediction

The prediction task used by Durschmid and colleagues (111) is a variation of the local-global paradigm, formed by two types of blocks, the predictable (P) and the unpredictable (U). The fully predictable blocks consist of the regular repetitions of the local-deviant (i.e., AAAAB AAAAB AAAAB …), where the location of the deviant tone (B) can be fully predicted (after the presentation of 4 As). In contrast, the local-deviant tone (B) does not follow any global rules in the unpredictable blocks. It appears between 3 and 7 repetitions of the local-standard tone (A). This design also allows the investigation of local and global rules hierarchically, fitting the scope of predictive coding.

Recent advancement of methodological (oddball paradigms) and conceptual (predictive coding) development in MMN has enabled us to better understand the functional and anatomical mechanism underlying it, namely, adaptation/deviance detection or hierarchical segmentation. This provides a valuable new perspective to the interpretation of existing neurological data on MMN and in generating hypotheses about the pathology of certain psychiatric disorders where MMN has long been used as a biomarker. Considered the widely diverse MMN findings and different pathologies associated with each psychiatric disorder, we decided to use one clinical condition as an example to illustrate how such new perspective could be applied to the existing literature and future studies. Schizophrenia has been chosen because the classic MMN, which is said to index the pre-attentive perceptual deficit, is perceived as a valid biomarker in schizophrenia.

## Mismatch Negativity in Schizophrenia

Reduced auditory MMN (104, 112–115), magnetic auditory MMN (116, 117) and visual MMN (118, 119) in a classic oddball paradigm is commonly observed on patients suffered from psychotic disorders. MMN also has the greatest effect size as a biomarker for schizophrenia amongst P50, N100, and P300 (120). Remarkably, there were significant associations between MMN amplitudes and psychotic symptom severity in cross-sectional comparisons (121–124), which inspired the possibility of using MMN amplitudes for clinical prognosis. Some research groups showed that an increase in MMN amplitude predicted the clinical remission of people with ultra-high risk for psychosis (UHR) (125, 126). Another longitudinal study revealed that MMN deficits might be associated with the negative symptoms and functional outcomes of the patients with schizophrenia (127). Unfortunately, these studies did not control medication dosage as well as other medical care and treatment as confounders, therefore the actual contribution of MMN can only be evaluated in larger longitudinal samples with appropriate confounding control.

Several studies have attempted to investigate the association between MMN amplitude and the trajectory of psychosis development: adolescent preclinical psychotic-like experiences (PLEs), UHR, first-episode psychosis (FEP), first-episode schizophrenia (FESZ), and chronic schizophrenia. Cross-sectional studies have shown that MMN amplitude evoked by duration deviants (dMMN) was signiﬁcantly reduced in earlier clinical stages of psychosis - UHR and FEP, compared to healthy controls, while such differences were not observed using frequency deviants (fMMN) (113, 121, 128–134). Reduced dMMN amplitude can also be observed on adolescents having PLEs (135) and people with UHR who later converted to psychosis (130). The attenuation of a double (duration + frequency) deviants evoked MMN also predicted the conversion of UHR to FEP (136). Collectively, dMMN attenuation can be observed across different stages of psychosis, making it a valuable trait marker.

However, the association between MMN and the development of psychosis are still lacking in support from longitudinal investigations. Some longitudinal findings revealed a progressive reduction of MMN amplitude in FESZ (137, 138) but not in chronic schizophrenia (139). Salisbury and colleagues also found a progressive reduction of fMMN alongside the volume decrease at the left Heschl’s gyrus for patients with schizophrenia instead of bipolar disorder and healthy controls (138). However, these findings are somewhat controversial with no group difference nor progressive reduction being seen between UHR, FEP, and control groups (115). A similar trend has been reported for dMMN (140) but not in others (115, 141). Whether this controversy originates from the difference in measurement procedure, recruitment, the definition of clinical stages or follow-up period remained unanswered.

Compared to schizophrenia, there were fewer examinations on MMN for other psychiatric disorders. Cross-disease comparisons have shown that patients with bipolar disorder had a decreased dMMN amplitude similar to those with schizophrenia compared to healthy controls (123). Another studied showed a contradictory finding in which their patients with bipolar disorder and major depressive disorder had no significant dMMN amplitude reduction (142). A review of the MMN study on the bipolar spectrum has proposed that bipolar disease could lead to an intermediate level reduction on MMN in-between those with schizophrenia and the healthy controls (143) although a systematic model is yet to be developed due to the unclear relationship between MMN and the subtypes (as well as medication status) of bipolar disorder (144, 145). MMN for the major depressive disorder had also been investigated, yet again, the results were inconsistent across the literatures (146–148).

Recent cross-disease multi-modal studies have proposed that the deficits in MMN amplitude could be driven by the structural change at specific brain regions rather than the disease pathology. This idea is supported by the intermediate dMMN amplitude decrease of patients with bipolar disorder compared to patients with schizophrenia and healthy controls, while the amplitude was associated with the cortical thickness of the right STG (149), suggesting that the MMN amplitude could rather indicate the deficit of higher-order auditory functions in the STG irrespective of the spectrum of the psychiatric disorders. A functional MRI study showed that the deviant-induced BOLD signal for patients with schizophrenia was reduced in a few cortical areas and subcortical areas (IC, thalamus, AC, and PFC) and those with major depressive disorder only had the reduced BOLD on the PFC (73). Some researchers argued that there could be broader pathophysiology for MMN (143), and therefore, MMN should not be used as a biomarker exclusively for schizophrenia. With our understanding of the formation of MMN using predictive coding, it appears that MMN can index different psychopathology that are shared across different psychotic and related disorders.

## Pathology of Schizophrenia From a Predictive Coding Perspective

The classification of most psychiatric disorders nowadays such as the Diagnostic and Statistical Manual (DSM-V) and International Classification of Diseases (ICD-10) diagnoses the presence or absence of a disorder based on a list of categorical symptoms. For example, schizophrenia is characterized by positive symptoms such as delusions and hallucinations.

Based on predictive coding theory, some researchers argued that exaggerating or overly precise prior beliefs renders a sensory noise into an organized and reasoned percept (150). Since the precision of the sensory data and the prior belief (memory) would both be inferred to the posterior (updated perception), a relatively high prior belief would reduce the influence of the sensory data on the resultant perception. A recent study showed support to this notion by building prior belief through the repeated presentation of a visual cue concurrently with an auditory tone (151). They demonstrated that it was easier for the visual cue (in the absence of the actual tone) to induce hallucinatory tones as well as activations in certain cortical areas, such as AC, posterior superior temporal sulcus and anterior cingulate cortex, on psychotic patients who have hallucination symptoms. However, this appears as contradictory to some early studies of schizophrenia, where patients are less susceptible to hollow-mask illusion (a concave mask creating an illusion of convex face) (152, 153). Using a dynamic causal model, the authors showed that patients with schizophrenia had a stronger feedforward modulation from the primary visual cortex to the lateral occipital complex, implying a weak inference from learned prior beliefs compared to the sensory input. Although the above two examples may highlight two completely different neuropathology, the over/under reliance on prior belief could co-exist through a failed prediction error feedback mechanism.

The failure of prediction error feedback could distort perceptions by disrupting both the top-down modulation and bottom-up sensory projection. Such deficit of patients with schizophrenia in prediction error was more clearly demonstrated by MMN studies using other forms of oddball paradigm. In a local-global oddball study, patients with schizophrenia showed reduced MMN to both the local and global deviant compared to healthy control (154). Although this paradigm could not distinguish repetition suppression and deviance detection from one to each other, it revealed that the prediction error deficit of schizophrenia covers both the lower-level and higher-level processing layer. The reduced MMN was also shown in another study using roving paradigm (109, 155). Interestingly, the patients’ MMN only become visible with increasing repetitions of the standard tones. With a longer sequence of standard, MMN is believed to be predominated by the “release” of SSA-sensitive N1, which suggested that the SSA function for schizophrenia remains intact. This finding is consistent with other studies using many-standard control and omission deviant, which revealed that the prediction error for schizophrenia was mainly influenced by deviance detection rather than repetition suppression (154, 156, 157).

Patients with schizophrenia also had a progressive volume reduction in STG (158), a site known to associate with deviance detection (97). Previous research has also revealed that MMN is associated with the N-methyl-D-aspartate glutamate (NMDA) system (159–162). For example, an animal study showed that ketamine as an NMDA-antagonist can suppress both the local- and global-deviant triggered MMNs (163). Such association between MMN and NMDA, however, is only found in the deviance detection process, but not adaptation (102). This appeared to be consistent with the NMDA receptor (NMDA) hypofunction model on schizophrenia, which proposed that the pathology of schizophrenia involved a dysfunction or a blockage of the NMDA receptor (164–168).

It is worth mentioning that the hyperactivity in the dopaminergic system is tied to positive symptoms of schizophrenia such as verbal hallucination and persecutory delusion (169, 170) since some of the positive symptoms can be induced by dopamine stimulants (e.g., methamphetamine administration) and most of the antipsychotics that act as D2 receptor (D2R) antagonists for positive symptoms. Aberrant reward response was often associated with the above positive symptoms; thus, the dysfunction and imbalance of D1 receptor (D1R) and D2R were presumably linked to reward prediction error deficit (171–174). Such deficit prevents our brain from updating and correcting the internal prediction model that is modulated by reward which leads to false or hallucinatory percepts (169).

Previous studies have hypothesized that the dopaminergic pathway in reward system relies on the valence coding function of D1R and D2R: the former is responsible for the positive, while the latter is for the negative valence (175). Considering the clinical pharmacological findings of D2R and the functions of serotonin, positive and depressive symptoms were once expressed as the imbalance of D1R and D2R functions, with serotonin as a modulator for dopamine signalling (176). However, a recent study found that during the learning process, D1R in the nucleus accumbens and D2R were responsible for the generalization and discrimination phrases respectively (177). The excessive D1 and insufficient D2 activation may lead to overgeneralization that results in false belief and delusions, whereas the opposite may inhibit beliefs and prediction.

Collectively, we demonstrated how the impaired function of prediction contributes to psychiatric disorder/symptoms using schizophrenia as an example from the predictive coding perspective. The deficit of deviance detection in psychotic disorder as shown in MMN studies indicated that psychotic symptoms could originate from the failed sensory prediction located at the higher cortical level. Such impairments could be associated with the dopaminergic system that would stop sensory cues to be encoded into experiences and beliefs (172), and thus disrupting the sensory prediction scheme, causing delusions or other positive symptoms.

## Conclusion

Predictive coding models perceive MMN as an indicator of neuronal prediction error in a hierarchical structure. Although this model may have overstated the actual neural responses of prediction error, it provides a simple working model for researchers to concisely examine the functional or anatomical impairment of different neuropsychiatric population. Given a large number of relevant studies, we believe that in the future of the field, efforts should be made to obtain knowledge with more in-depth “details” instead of staying with its “simplicity” and “elegance”. Since prediction and prediction error were hypothesized to dynamically interact at each hierarchy, an obvious future direction could focus on the prediction signal. This could be achieved by isolating the adaptation mechanism from MMN using the classical oddball paradigm in combination with the many-standard control and cascadic control in which prediction error is minimized. More importantly, researchers seemed to accept the logic of neural arithmetics (i.e., prediction error = sensory input - prediction), despite the fact that how such computation could be carried out at each neuronal level are completely unknown. Considered the cost of monitoring the neural activities at the cellular level, refining and utilizing oddball paradigms such as local-global or prediction paradigm to achieve hierarchal segmentation appears as a sensible solution to unveil the full picture of sensory prediction.

## Author Contributions

CF and WL contributed to writing the draft manuscript. TU and SK contributed to conceptualization of this review and reviewing the manuscript. All authors contributed to the article and approved the submitted version.

## Funding

This research was supported by the Agency for Medical Research and Development (AMED) under grant numbers JP20dm0207069 and by the Japan Society for the Promotion of Science (JSPS) KAKENHI grant numbers 19H03579 and 19H04878. This study was also supported by the University of Tokyo Center for Integrative Science of Human Behavior (CiSHuB) and the International Research Center for Neurointelligence (WPI-IRCN) at the University of Tokyo Institutes for Advanced Study (UTIAS).

## Conflict of Interest

The authors declare that the research was conducted in the absence of any commercial or financial relationships that could be construed as a potential conflict of interest.
